# Beyond the Sin-G family: The transformed Sin-G family

**DOI:** 10.1371/journal.pone.0250790

**Published:** 2021-05-11

**Authors:** Farrukh Jamal, Christophe Chesneau, Dalal Lala Bouali, Mahmood Ul Hassan

**Affiliations:** 1 Department of Statistics, The Islamia University, Bahawalpur, Pakistan; 2 Department of Mathematics, Université de Caen, Caen, France; 3 Laboratory of Applied Mathematics, Mohamed Khider University of Biskra, Biskra, Algeria; 4 Stockholm University, Stockholm, Sweden; Roswell Park Cancer Institute, UNITED STATES

## Abstract

In recent years, the trigonometric families of continuous distributions have found a place of choice in the theory and practice of statistics, with the Sin-G family as leader. In this paper, we provide some contributions to the subject by introducing a flexible extension of the Sin-G family, called the transformed Sin-G family. It is constructed from a new polynomial-trigonometric function presenting a desirable “versatile concave/convex” property, among others. The modelling possibilities of the former Sin-G family are thus multiplied. This potential is also highlighted by a complete theoretical work, showing stochastic ordering results, studying the analytical properties of the main functions, deriving several kinds of moments, and discussing the reliability parameter as well. Then, the applied side of the proposed family is investigated, with numerical results and applications on the related models. In particular, the estimation of the unknown model parameters is performed through the use of the maximum likelihood method. Then, two real life data sets are analyzed by a new extended Weibull model derived to the considered trigonometric mechanism. We show that it performs the best among seven comparable models, illustrating the importance of the findings.

## 1 Introduction

Recent advances in probability distribution theory and applications have seen the rise of various general families of distributions, successfully applied for different statistical problems. In this regard, a nice survey can be found in [1]. Here, we put the light on the trigonometric families of continuous distributions, i.e., those defined by a cumulative distribution function (cdf) involving trigonometric functions (sine, cosine, tangent, cotangent, and various combinations of these). The pioneer work is about the Sin-G family developed by [2–5]. As indicated by its name, it is defined around the sine function; the corresponding cdf is given by

$$\begin{array}{c} F\left(x;\zeta \right)=\sin[\frac{\pi }{2}G\left(x;\zeta \right)],\;x\in R, \end{array}$$ (1)

where *G*(*x*;*ζ*) is a baseline cdf of a continuous distribution with parameter(s) vector denoted by *ζ*. It is now demonstrated that the Sin-G family has the ability to provide flexible statistical models to fit data of various nature. Also, it is a simple alternative to the model derived to the baseline distribution, without the addition of parameter. For instance, in [2], the exponential distribution is used as a baseline to construct the SinE model, which reveals to suitably fit the famous bladder cancer patients data of [6]. Also, he has the better fit as compared to some classical models such as the former exponential one, having better Akaike information criteria (AIC), Bayesian information criteria (BIC) and Kolmogorov-Smirnov (KS) test values. On the other side, based on the inverse Weibull distribution (see [7]), the SinIW model was introduced by [4], with application to the so-called Guinea pigs data by [8], providing better BIC in comparison to some other solid models. A“free for all” R package on the SinIW model is provided in [9]. As a matter of fact, the qualities of the models derived to the Sin-G family have inspired other general families of continuous distributions also centered around trigonometric functions, such as the Cos-G family by [5], CS-G family by [10], NSin-G family by [11], TransSC-G family by [12], SinTL-G family by [13], SinKum-G family by [14], and SinEOF-G family by [15]. The majority of these families are based on the Sin-G structure, with no additional tuning parameters or transformations.

In this paper, we go further the Sin-G family by proposing a new extended version of it, called the transformed Sin-G (TS-G) family. The corresponding cdf is derived to (1), with the use of a simple one-parameter polynomial-trigonometric transformation. This transformation has the following features: (i) it is analytically simple and includes the non-transformed case, (ii) it has the properties of a continuous cdf, that is, has its values into the unit interval, is continuous, almost everywhere differentiable and increasing, and (iii) it can be convex or concave, or none of them, for well-identified values of the parameter. Thanks to its versatility, this transformation significantly enhances the flexible properties of (1), and the baseline cdf as well. Thus, the TS-G family distinguishes itself from other modified Sin-G families by its overall simplicity, original polynomial-trigonometric functions, and the advantage of flexible kurtosis, skewness, versatile distribution tails, and various hazard rate shapes, as a result of the considered transformation. Thus, the TS-G family can provide interesting models for diverse fitting purposes. This practical aspect, along with important theoretical results, are developed in this study.

The rest of the paper is organized as follows. The basics on the TS-G family are presented in Section 2. Also, an emphasis is put on a special distribution of the family based on the Weibull distribution, motivated by its desirable shapes characteristics in the modelling sense. In Section 3, interesting properties of the TS-G family are studied, including stochastic ordering results, equivalence properties, critical points analysis, series expansion involving known exponentiated functions, moments, and reliability parameter. In Section 4, by adopting a statistical approach, the TS-G model parameters are estimated with the maximum likelihood method, supported by a simulation study. Then, applications of this special model are addressed in Section 5, showing how the new family can be of interest to fit various data sets, outperforming seven other solid extended or modified Weibull models of the literature. Section 6 formulates concluding remarks.

## 2 Basics on the TS-G family

In this section, the TS-G family is defined, with motivations and discussions.

### 2.1 On a special polynomial-trigonometric function

The following result presents some interesting features of a simple polynomial-trigonometric function, which will be at the basis of the TS-G family.

**Proposition 1** *Let* λ ∈ [0, 1] *and T*_λ_(*x*) *be the following parametric function*:

$$\begin{array}{c} T_{\lambda }\left(x\right)=\sin(\frac{\pi }{2}x)-\lambda \frac{\pi }{2}x\cos(\frac{\pi }{2}x),\;x\in \left[0,1\right], \end{array}$$ (2)

*with T*_λ_(*x*) = 0 *if x* < 0 *and T*_λ_(*x*) = 1 *if x* > 1. *Then, the following properties hold*:

- *T*_λ_(*x*) *has the properties of a continuous cdf*,
- *T*_λ_(*x*) *can be convex or concave according to the values of* λ. *In particular, for* λ ∈ [0, 1/3], *T*_λ_(*x*) *is concave and, for* λ ∈ [1/2, 1], *T*_λ_(*x*) *is convex*.
- *For* λ ∈ (1/3, 1/2), *T*_λ_(*x*) *can be neither convex nor concave*.

**Proof**. First of all, the following inequality holds: for *y* ∈ [0, *π*/2], we have

$$\begin{array}{c} \sin(y)\geq y\cos(y), \end{array}$$ (3)

(see [16]). Let us now prove the first point of the proposition. Since λ ∈ [0, 1], it follows from (3) that 0 ≤ *T*_1_(*x*)≤*T*_λ_(*x*)≤sin[(*π*/2)*x*] ≤ 1. Also, *T*_λ_(*x*) satisfies *T*_λ_(0) = 0 and *T*_λ_(1) = 1, it is continuous, differentiable and, by differentiating on *x*, we have

$$\begin{array}{c} \frac{d}{dx}T_{\lambda }\left(x\right)=\frac{\pi }{2}[\left(1-\lambda \right)\cos(\frac{\pi }{2}x)+\lambda \frac{\pi }{2}x\sin(\frac{\pi }{2}x)]. \end{array}$$

As a sum of positive functions, we have *dT*_λ_(*x*)/*dx* ≥ 0, so *T*_λ_(*x*) is increasing. We conclude that *T*_λ_(*x*) has the properties of a continuous cdf. For the second point of the proof, let us notice that, by differentiating on *x*, we have

$$\begin{array}{c} \frac{d^{2}}{dx^{2}}T_{\lambda }\left(x\right)=\frac{\pi^{2}}{4}[\left(2\lambda -1\right)\sin(\frac{\pi }{2}x)+\lambda \frac{\pi }{2}x\cos(\frac{\pi }{2}x)]. \end{array}$$

Therefore, if λ ∈ [0, 1/3], it follows from 2λ − 1 ≤ −1/3 and (3) that

$$\begin{array}{c} \frac{d^{2}}{dx^{2}}T_{\lambda }\left(x\right)\leq -\frac{\pi^{2}}{12}[\sin(\frac{\pi }{2}x)-\frac{\pi }{2}x\cos(\frac{\pi }{2}x)]\leq 0. \end{array}$$

That is, *T*_λ_(*x*) is concave. On the other hand, if λ ∈ [1/2, 1], we have *d*^2^ *T*_λ_(*x*)/*dx*^2^ ≥ 0 as a sum of positive functions, implying that *T*_λ_(*x*) is convex.

**Fig 1:**
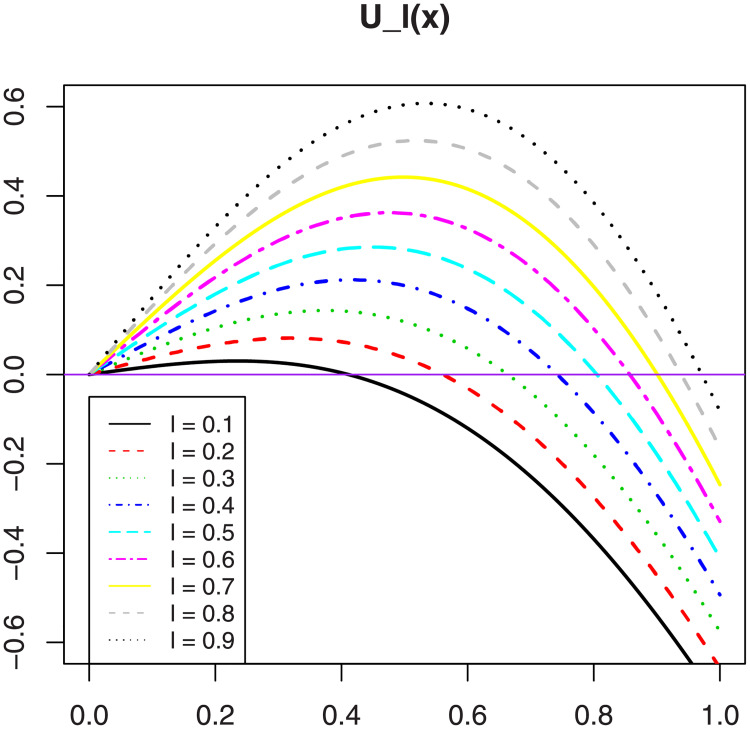
Plots of the function *U*_*ℓ*_(*x*) for *ℓ* ∈ {0.1, 0.2, …, 0.9}.

Now, for λ = 2/5 ∈ (1/3, 1/2), we have

$$\begin{array}{c} \frac{d^{2}}{dx^{2}}T_{\lambda }\left(0.1\right)=0.07592538>0,\;\frac{d^{2}}{dx^{2}}T_{\lambda }\left(0.8\right)=-0.08606892<0, \end{array}$$

implying that *T*_λ_(*x*) can be neither convex nor concave. As a visual approach, if we set $U_{\ell }\left(x\right)=d^{2}T_{\lambda_{\ell }}\left(x\right)/dx^{2}$, with λ_*ℓ*_ = *ℓ*/2 + (1 − *ℓ*)/3 and *ℓ* ∈ {0.1, 0.2, …, 0.9}, so that λ_*ℓ*_ ∈ (1/3, 1/2), Fig 1 shows that *U*_*ℓ*_(*x*) can be positive and negative, implying that $T_{\lambda }\left(x\right)$ is neither convex nor concave for the considered values of λ. This concludes the proof of Proposition 1.

One can remark that the function *T*_λ_(*x*) defined by (2) can be written as $T_{\lambda }\left(x\right)=T_{\lambda }^{*}\{\sin[(\pi /2)x]\}$, where

$$\begin{array}{c} T_{\lambda }^{*}\left(x\right)=x-\lambda \arcsin\left(x\right)\sqrt{1-x^{2}},\;x\in \left[0,1\right]. \end{array}$$

One can establish that the function $T_{\lambda }^{*}\left(x\right)$ has the properties of a cdf, which is not mentioned in the existing literature.

**Fig 2:**
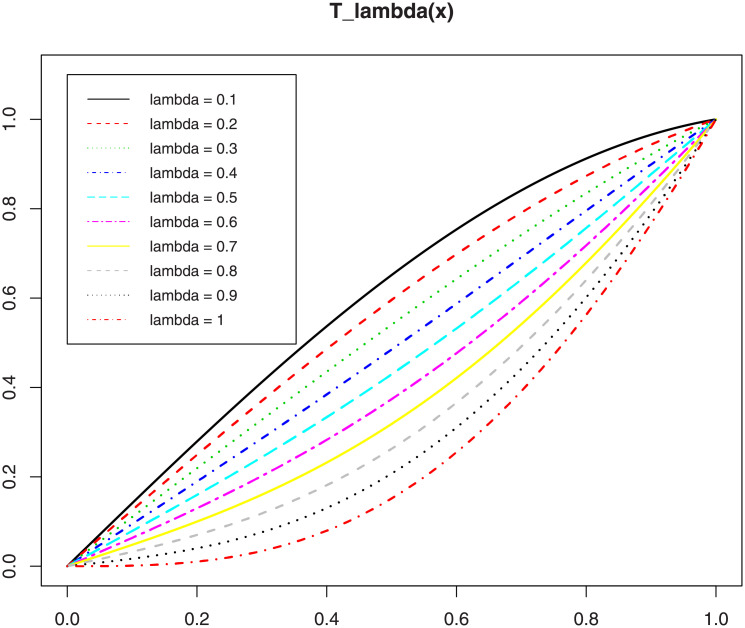
Plots of the function *T*_λ_(*x*) for λ ∈ {0.1, 0.2, …, 1}.

In view of Proposition 1, the transformation function $T_{\lambda }^{*}\left(x\right)$ allows to “convexify (or not)” the convex cdf *s*(*x*) = sin[(*π*/2)*x*], *x* ∈ [0, 1], while keeping its cdf properties. This ability is not satisfied by some other simple transformation functions, as the power transformation, i.e., $T_{\gamma }^{**}\left(x\right)=x^{\gamma }$ with *γ* > 0, for instance. This aspect is the driving force behind the TS-G family, which aims to expand the Sin-G family in a straightforward manner to open new statistical perspectives. We show the convex/concave properties of the function *T*_λ_(*x*) given by (2) in Fig 2, by considering several values for λ.

### 2.2 Definition

By taking the benefits of the flexibility of *T*_λ_(*x*) given by (2) as described in Proposition 1, the proposed TS-G family of continuous distributions is defined by the following cdf:

$$\begin{array}{c} F\left(x;\lambda ,\zeta \right)=\sin[\frac{\pi }{2}G\left(x;\zeta \right)]-\lambda \frac{\pi }{2}G\left(x;\zeta \right)\cos[\frac{\pi }{2}G\left(x;\zeta \right)],\;x\in R, \end{array}$$ (4)

where λ ∈ [0, 1] and, as usual, *G*(*x*;*ζ*) is a baseline cdf of a continuous distribution with parameter(s) vector denoted by *ζ*.

That is, by considering the transformations *T*_λ_(*x*) and $T_{\lambda }^{*}\left(x\right)$ discussed above, we have *F*(*x*;λ, *ζ*) = *T*_λ_[*G*(*x*;*ζ*)] or, equivalently, $F\left(x;\lambda ,\zeta \right)=T_{\lambda }^{*}\{\sin[(\pi /2)G(x;\zeta )]\}$, motivating the name of “transformed Sin-G family”. One can notice that the cdf of the former Sin-G family is derived by taking λ = 0. Also, based on Proposition 1 and the convex/concave properties of $T_{\lambda }^{*}\left(x\right)$, we argue that the overall flexibility of the cdf of the former Sin-G family provided by (1) is enhanced. This is concretized by the addition of the modulating polynomial-cosine term λ(*π*/2)*G*(*x*;*ζ*)cos[(*π*/2)*G*(*x*;*ζ*)], which opens up a whole new world of possibilities.

Also, one can write *F*(*x*;λ, *ζ*) as a simple mixture of two cdfs of the TS-G family itself: *F*(*x*;0, *ζ*) and *F*(*x*;1, *ζ*), with the weights 1 − λ and λ, respectively, i.e.,

$$\begin{array}{c} F(x;\lambda ,\zeta )=(1-\lambda )F(x;0,\zeta )+\lambda F(x;1,\zeta ). \end{array}$$

Hence, the role of λ is to balance *F*(*x*;0, *ζ*) and *F*(*x*;1, *ζ*), each reaching different targets in terms of statistical modelling.

Among the other functions of interest, the survival function (sf) of the TS-G family is given by

$$\begin{array}{c} S\left(x;\lambda ,\zeta \right)=1-\sin[\frac{\pi }{2}G\left(x;\zeta \right)]+\lambda \frac{\pi }{2}G\left(x;\zeta \right)\cos[\frac{\pi }{2}G\left(x;\zeta \right)],\;x\in R. \end{array}$$

Upon an almost everywhere differentiation of *F*(*x*;λ, *ζ*) with respect to *x*, the corresponding probability density function (pdf) is given by

$$\begin{array}{c} f\left(x;\lambda ,\zeta \right)=\frac{\pi }{2}g\left(x;\zeta \right)\{\lambda \frac{\pi }{2}G\left(x;\zeta \right)\sin[\frac{\pi }{2}G\left(x;\zeta \right)]+\left(1-\lambda \right)\cos[\frac{\pi }{2}G\left(x;\zeta \right)]\}, \end{array}$$ (5)

where *g*(*x*;*ζ*) is the pdf of the baseline distribution, i.e., obtained by an almost everywhere differentiation of *G*(*x*;*ζ*).

Another important function of the TS-G family, specially when the support of the baseline distribution is (0, + ∞), is the hazard rate function (hrf) defined by

$$\begin{array}{c} h\left(x;\lambda ,\zeta \right)=\frac{\frac{\pi }{2}g\left(x;\zeta \right)\{\lambda \frac{\pi }{2}G\left(x;\zeta \right)\sin[\frac{\pi }{2}G\left(x;\zeta \right)]+\left(1-\lambda \right)\cos[\frac{\pi }{2}G\left(x;\zeta \right)]\}}{1-\sin[\frac{\pi }{2}G\left(x;\zeta \right)]+\lambda \frac{\pi }{2}G\left(x;\zeta \right)\cos[\frac{\pi }{2}G\left(x;\zeta \right)]},\;x\in R. \end{array}$$ (6)

For the importance of the sf and hrf, in reliability analysis mainly, we may refer the reader to [17], and the references therein.

### 2.3 A special distribution: The TSW distribution

Naturally, each choice for *G*(*x*;*ζ*) gives a new TS-G distribution. Here, we focus our attention on the Weibull distribution as baseline, i.e., defined by the following cdf:

$$\begin{array}{c} G\left(x;\alpha ,\beta \right)=1-e^{-\alpha x^{\beta }},\;x>0, \end{array}$$ (7)

and *G*(*x*;*α*, *β*) = 0 if *x* ≤ 0, where *α* > 0 and *β* > 0 are scale and shape parameters, respectively. As a main interest, the Weibull distribution is known to be an alternative to the exponential distribution, offering more flexible hazard rate shapes; decreasing and increasing shapes can be observed. It has been involved with success in a plethora of applications requiring the analysis of lifetime and reliability data. In this regard, we may refer the reader to [18–20].

We thus aim to extend the Weibull distribution, along with their properties, via the use of the TS-G family. That is, by inserting (7) into (4), we introduce the TSW distribution defined by the following cdf:

$$\begin{array}{cc} F(x;\lambda ,\alpha ,\beta ) & =\sin[\frac{\pi }{2}\left(1-e^{-\alpha x^{\beta }}\right)]-\lambda \frac{\pi }{2}\left(1-e^{-\alpha x^{\beta }}\right)\cos[\frac{\pi }{2}\left(1-e^{-\alpha x^{\beta }}\right)]\\ & =\cos[\frac{\pi }{2}e^{-\alpha x^{\beta }}]-\lambda \frac{\pi }{2}\left(1-e^{-\alpha x^{\beta }}\right)\sin[\frac{\pi }{2}e^{-\alpha x^{\beta }}],\;x>0, \end{array}$$

and *F*(*x*;λ, *α*, *β*) = 0 if *x* ≤ 0, where the second expression is obtained after some trigonometric manipulations.

Also, the corresponding sf, pdf and hrf are, respectively, given by

$$\begin{array}{c} S\left(x;\lambda ,\alpha ,\beta \right)=1-\cos[\frac{\pi }{2}e^{-\alpha x^{\beta }}]+\lambda \frac{\pi }{2}\left(1-e^{-\alpha x^{\beta }}\right)\sin[\frac{\pi }{2}e^{-\alpha x^{\beta }}],\;x>0, \end{array}$$

and *S*(*x*;λ, *α*, *β*) = 1 if *x* ≤ 0,

$$\begin{array}{cc} & f\left(x;\lambda ,\alpha ,\beta \right)=\frac{\pi }{2}\alpha \beta x^{\beta -1}e^{-\alpha x^{\beta }}\{\lambda \frac{\pi }{2}\left(1-e^{-\alpha x^{\beta }}\right)\cos[\frac{\pi }{2}e^{-\alpha x^{\beta }}]+\left(1-\lambda \right)\sin[\frac{\pi }{2}e^{-\alpha x^{\beta }}]\},\\ & x>0, \end{array}$$

and *f*(*x*;λ, *α*, *β*) = 0 if *x* ≤ 0, and

$$\begin{array}{cc} & h\left(x;\lambda ,\alpha ,\beta \right)=\frac{\frac{\pi }{2}\alpha \beta x^{\beta -1}e^{-\alpha x^{\beta }}\{\lambda \frac{\pi }{2}\left(1-e^{-\alpha x^{\beta }}\right)\cos[\frac{\pi }{2}e^{-\alpha x^{\beta }}]+\left(1-\lambda \right)\sin[\frac{\pi }{2}e^{-\alpha x^{\beta }}]\}}{1-\cos[\frac{\pi }{2}e^{-\alpha x^{\beta }}]+\lambda \frac{\pi }{2}\left(1-e^{-\alpha x^{\beta }}\right)\sin[\frac{\pi }{2}e^{-\alpha x^{\beta }}]},\\ & x>0, \end{array}$$

and *h*(*x*;λ, *α*, *β*) = 0 if *x* ≤ 0.

**Fig 3:**
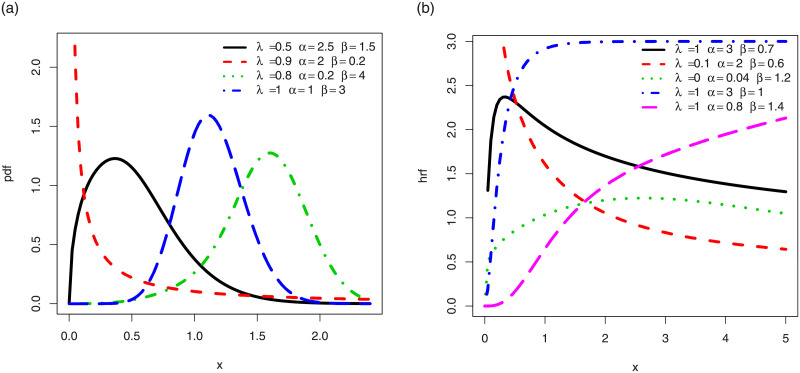
Selection of plots for (a) the pdf and (b) the hrf of the TSW distribution.

After some graphical investigations, the curvature properties of the functions of the TSW distribution reveal to be desirably versatile. Evidence can be seen in Fig 3, which displays some plots of the corresponding pdf and hrf for various values of the parameters.

In particular, Fig 3(a) indicates that the pdf of the TSW distribution has various skewness shapes (near symmetrical, left, right, bathtub, reversed-J shapes, mainly), along with different kurtosis properties. Fig 3(b) reveals that the corresponding hrf possesses versatile shapes, such as decreasing, increasing, bathtub (classic and upside-down) and reversed-J shapes. These observations imply that the TSW distribution is adequate to fit heterogeneous data sets. In our study, this aspect will be developed in Section 5, where the TSW distribution is used to fit two real life data sets. Also, it will be compared with other extended or modified Weibull models, and the results will be quite favorable to the TSW model.

## 3 Notable mathematical properties

Here, we explore some mathematical properties of interest satisfied by the TS-G family.

### 3.1 Stochastic ordering results

Stochastic ordering results are crucial to understand a certain hierarchy existing between the distributions, with consequence on their comparison from the modelling point of view. In the framework of the TS-G family, the following result presents some relations involving the cdf of the TS-G family (beyond the following immediate stochastic ordering property: *F*(*x*;λ, *ζ*)≤*F*(*x*;0, *ζ*)).

**Proposition 2** *The following inequalities hold*:

- *If* λ_2_ ≥ λ_1_ ≥ 0, *we have F*(*x*;λ_2_, *ζ*)≤*F*(*x*;λ_1_, *ζ*).
- *For* λ ∈ [0, 2/*π*], *we have F*(*x*;λ, *ζ*)≥F_*_(*x*;*ζ*), *where*
  $$\begin{array}{c} F_{*}\left(x;\zeta \right)=G\left(x;\zeta \right)\{1-\cos[\frac{\pi }{2}G\left(x;\zeta \right)]\} \end{array}$$
  *is a valid cdf*.

**Proof**. Based on (4), since λ_2_ ≥ λ_1_ and the involved functions are positive, we have

$$\begin{array}{c} \sin[\frac{\pi }{2}G\left(x;\zeta \right)]-\lambda_{2}\frac{\pi }{2}G\left(x;\zeta \right)\cos[\frac{\pi }{2}G\left(x;\zeta \right)]\leq \sin[\frac{\pi }{2}G\left(x;\zeta \right)]-\lambda_{1}\frac{\pi }{2}G\left(x;\zeta \right)\cos[\frac{\pi }{2}G\left(x;\zeta \right)], \end{array}$$

implying the desired inequality.

For the second point, the following inequality holds: for *y* ∈ [0, *π*/2], we have sin(*y*)≥*y*(2/*π*) (see [16]). Hence, based on (4), since λ ∈ [0, 2/*π*], we have

$$\begin{array}{cc} F(x;\lambda ,\zeta ) & \geq G\left(x;\zeta \right)-\lambda \frac{\pi }{2}G\left(x;\zeta \right)\cos[\frac{\pi }{2}G\left(x;\zeta \right)]\\ & \geq G\left(x;\zeta \right)-G\left(x;\zeta \right)\cos[\frac{\pi }{2}G\left(x;\zeta \right)]=F_{*}\left(x;\zeta \right). \end{array}$$

Then, one can remark that F_*_(*x*;*ζ*) is the cdf of the rv *Z* = max(*X*, *Y*), where *X* is a rv having the (baseline) cdf *G*(*x*;*ζ*) and *Y* is a rv having the cdf of the Cos-G family (see [5]), with *X* and *Y* independent.

The following result is about a likelihood stochastic ordering of the TS-G family. We refer the reader to [21] for the details on the concept of likelihood stochastic order.

**Proposition 3** *Let X*_1_ *be a rv having the cdf F*(*x*;λ_1_, *ζ*) *and X*_2_ *be a rv having the cdf F*(*x*;λ_2_, *ζ*). *Then, if* λ_2_ ≥ λ_1_, *we have X*_1_ ≤ *X*_2_ *in the likelihood stochastic ordering sense*.

**Proof**. Following [21], we have *X*_1_ ≤ *X*_2_ in the likelihood stochastic ordering sense if and only if the following ratio function is decreasing with respect to *x*:

$$\begin{array}{c} r\left(x;\lambda_{1},\lambda_{2},\zeta \right)=\frac{f(x;\lambda_{1},\zeta )}{f(x;\lambda_{2},\zeta )},\;x\in R, \end{array}$$

where *f*(*x*;λ_1_, *ζ*) and *f*(*x*;λ_2_, *ζ*) are the corresponding pdfs of *F*(*x*;λ_1_, *ζ*) and *F*(*x*;λ_2_, *ζ*), respectively. That is, by using (5), we have

$$\begin{array}{c} r\left(x;\lambda_{1},\lambda_{2},\zeta \right)=\frac{\lambda_{1}\frac{\pi }{2}G\left(x;\zeta \right)\sin[\frac{\pi }{2}G\left(x;\zeta \right)]+\left(1-\lambda_{1}\right)\cos[\frac{\pi }{2}G\left(x;\zeta \right)]}{\lambda_{2}\frac{\pi }{2}G\left(x;\zeta \right)\sin[\frac{\pi }{2}G\left(x;\zeta \right)]+\left(1-\lambda_{2}\right)\cos[\frac{\pi }{2}G\left(x;\zeta \right)]},\;x\in R. \end{array}$$

Upon an almost everywhere differentiation with respect to *x*, after some developments, we get

$$\begin{array}{c} \frac{d}{dx}r\left(x;\lambda_{1},\lambda_{2},\zeta \right)=\left(\lambda_{1}-\lambda_{2}\right)\frac{\pi g\left(x;\zeta \right)[\pi G\left(x;\zeta \right)+\sin[\pi G(x;\zeta )]]}{4{\left\{\lambda_{2}\frac{\pi }{2}G\left(x;\zeta \right)\sin[\frac{\pi }{2}G\left(x;\zeta \right)]+\left(1-\lambda_{2}\right)\cos[\frac{\pi }{2}G\left(x;\zeta \right)]\right\}}^{2}}, \end{array}$$

which is negative if and only if λ_2_ ≥ λ_1_, implying the desired result.

### 3.2 Equivalence properties

Here, some equivalence properties of crucial functions of the TS-G family are discussed, which can be helpful to find their limits and also, understand the tails properties of the distribution. As *G*(*x*;*ζ*)→0, we establish that

$$\begin{array}{c} F\left(x;\lambda ,\zeta \right)∼\frac{\pi }{2}\left(1-\lambda \right)G\left(x;\zeta \right),\;f\left(x;\lambda ,\zeta \right)∼\frac{\pi }{2}\left(1-\lambda \right)g\left(x;\zeta \right),\;h\left(x;\lambda ,\zeta \right)∼\frac{\pi }{2}\left(1-\lambda \right)g\left(x;\zeta \right). \end{array}$$

Also, as *G*(*x*;*ζ*)→1, we have

$$\begin{array}{c} F\left(x;\lambda ,\zeta \right)∼1-\frac{\pi^{2}}{4}\lambda \left[1-G\left(x;\zeta \right)\right],\;f\left(x;\lambda ,\zeta \right)∼\frac{\pi^{2}}{4}\lambda g\left(x;\zeta \right),\;h\left(x;\lambda ,\zeta \right)∼\frac{g(x;\zeta )}{1-G(x;\zeta )}. \end{array}$$

In each case, we see how the new parameter λ modulates the limits; it has a strong effect in this regard, except for the hrf when *G*(*x*;*ζ*)→1.

In the case of the TSW distribution as described in Subsection 2.3, the following equivalence hold. As *x* → 0, we have

$$\begin{array}{c} F\left(x;\lambda ,\alpha ,\beta \right)∼\frac{\pi }{2}\left(1-\lambda \right)\alpha x^{\beta },\;f\left(x;\lambda ,\alpha ,\beta \right)∼\frac{\pi }{2}\left(1-\lambda \right)\alpha \beta x^{\beta -1} \end{array}$$

and

$$\begin{array}{c} h\left(x;\lambda ,\alpha ,\beta \right)∼\frac{\pi }{2}\left(1-\lambda \right)\alpha \beta x^{\beta -1}. \end{array}$$

Therefore, we obtain lim_*x* → 0_ *f*(*x*;λ, *α*, *β*) = lim_*x* → 0_ *f*(*x*;λ, *α*, *β*) = *ℓ* with *ℓ* = + ∞ if *β* < 1, *ℓ* = (*π*/2)(1 − λ)*α* if *β* = 1 and *ℓ* = 0 if *β* > 1.

Also, as *x* → + ∞, we have

$$\begin{array}{c} F\left(x;\lambda ,\alpha ,\beta \right)∼1-\frac{\pi^{2}}{4}\lambda e^{-\alpha x^{\beta }},\;f\left(x;\lambda ,\alpha ,\beta \right)∼\frac{\pi^{2}}{4}\lambda \alpha \beta x^{\beta -1}e^{-\alpha x^{\beta }} \end{array}$$

and

$$\begin{array}{c} h\left(x;\lambda ,\alpha ,\beta \right)∼\alpha \beta x^{\beta -1}. \end{array}$$

Hence, we have lim_*x* → + ∞_ *f*(*x*;λ, *α*, *β*) = 0 in all the situations, and lim_*x* → + ∞_ *h*(*x*;λ, *α*, *β*) = *ℓ* with *ℓ* = 0 if *β* < 1, *ℓ* = *α* if *β* = 1 and *ℓ* = + ∞ if *β* > 1.

### 3.3 Critical points

Some analytical facts about the critical points of functions of the TS-G family are now presented. First of all, the study of critical point(s), i.e., mode(s), of *f*(*x*;λ, *ζ*) informs us on the possible singularities of the related model. A critical point of *f*(*x*;λ, *ζ*), say x_*_, is solution of the following non-linear equation: *df*(*x*;λ, *ζ*)/*dx* = 0, which is equivalent to be solution of the following more tractable non-linear equation: *d*{log[*f*(*x*;λ, *ζ*)]}/*dx* = 0, i.e.,

$$\begin{array}{c} \frac{dg(x;\zeta )/dx}{g(x;\zeta )}+\frac{\pi }{2}g\left(x;\zeta \right)\frac{\left(2\lambda -1\right)\sin[\frac{\pi }{2}G\left(x;\zeta \right)]+\frac{\pi }{2}\lambda G\left(x;\zeta \right)\cos[\frac{\pi }{2}G\left(x;\zeta \right)]}{\lambda \frac{\pi }{2}G\left(x;\zeta \right)\sin[\frac{\pi }{2}G\left(x;\zeta \right)]+\left(1-\lambda \right)\cos[\frac{\pi }{2}G\left(x;\zeta \right)]}=0. \end{array}$$

Then, the nature of x_*_ depends on the values of $\eta =d^{2}\left\{\log\left[f\left(x;\lambda ,\zeta \right)\right]\right\}/dx^{2}{|}_{x=x_{*}}$. More specifically, x_*_ is designated as a local maximum point if *η* < 0, an inflection point if *η* = 0, and a local minimum point if *η* > 0.

The same methodology can be applied to study the critical points for *h*(*x*;λ, *ζ*), which can be useful to identify specific hazard rate shapes (monotonic, bathtub, S…) for a modelling aim. Let us just mention that a critical point for *h*(*x*;λ, *ζ*) is solution of the following non-linear equation: *d*{log[*h*(*x*;λ, *ζ*)]}/*dx* = 0, i.e.,

$$\begin{array}{cc} & \frac{dg(x;\zeta )/dx}{g(x;\zeta )}+\frac{\pi }{2}g\left(x;\zeta \right)\frac{\left(2\lambda -1\right)\sin[\frac{\pi }{2}G\left(x;\zeta \right)]+\frac{\pi }{2}\lambda G\left(x;\zeta \right)\cos[\frac{\pi }{2}G\left(x;\zeta \right)]}{\lambda \frac{\pi }{2}G\left(x;\zeta \right)\sin[\frac{\pi }{2}G\left(x;\zeta \right)]+\left(1-\lambda \right)\cos[\frac{\pi }{2}G\left(x;\zeta \right)]}\\ & +\frac{\frac{\pi }{2}g\left(x;\zeta \right)\{\lambda \frac{\pi }{2}G\left(x;\zeta \right)\sin[\frac{\pi }{2}G\left(x;\zeta \right)]+\left(1-\lambda \right)\cos[\frac{\pi }{2}G\left(x;\zeta \right)]\}}{1-\sin[\frac{\pi }{2}G\left(x;\zeta \right)]+\lambda \frac{\pi }{2}G\left(x;\zeta \right)\cos[\frac{\pi }{2}G\left(x;\zeta \right)]}=0. \end{array}$$

Clearly, in most of the cases, the critical points of *f*(*x*;λ, *ζ*) and *h*(*x*;λ, *ζ*) have not closed-forms. They can however be determined as numerical values by using mathematical softwares, as Mathematica, Python, R, Maltlab…

Concerning the TSW distribution, numerical investigations, supported by Fig 3 as well, show that it is unimodal, with a corresponding hrf that can have one critical point.

### 3.4 A series expansion

The following result establishes a new representation of the pdf of the TS-G family involving exponentiated baseline pdfs. Such results are common for the pdfs of modern general families of continuous distributions (see, e.g., [4, 11, 22]).

**Proposition 4** *For any x such that G*(*x*;*ζ*)<1, *the following series expansion holds*:

$$\begin{array}{c} f\left(x;\lambda ,\zeta \right)=\sum_{k=0}^{+\infty }a_{k}\upsilon_{2k+1}\left(x;\zeta \right), \end{array}$$

*where a*_*k*_ = (*π*/2)^2*k*+1^(−1)^*k*^[1 − λ(2*k* + 1)]/(2*k* + 1)! *and υ*_*γ*_ = *γg*(*x*;*ζ*)*G*(*x*;*ζ*)^*γ*−1^, *with γ* = 2*k* + 1.

**Proof**. Owing to the series expansions of the sine and cosine functions, after some developments, we get

$$\begin{array}{cc} F(x;\lambda ,\zeta ) & =\sin[\frac{\pi }{2}G\left(x;\zeta \right)]-\lambda \frac{\pi }{2}G\left(x;\zeta \right)\cos[\frac{\pi }{2}G\left(x;\zeta \right)],\\ & =\sum_{k=0}^{+\infty }\frac{{\left(-1\right)}^{k}}{(2k+1)!}{\left[\frac{\pi }{2}G\left(x;\zeta \right)\right]}^{2k+1}-\lambda \frac{\pi }{2}G\left(x;\zeta \right)\sum_{k=0}^{+\infty }\frac{{\left(-1\right)}^{k}}{(2k)!}{\left[\frac{\pi }{2}G\left(x;\zeta \right)\right]}^{2k}\\ & =\sum_{k=0}^{+\infty }a_{k}{\left[G(x;\zeta )\right]}^{2k+1}. \end{array}$$

We end the proof of Proposition 4 by differentiating the above function with respect to *x*.

Proposition 4 is of interest because the properties of most of the exponentiated standard distributions are well known, and thus, can be used to determine those of the TS-G family. Also, from the practical point of view, it allows us to define some integral terms by the means of (infinite) sums, which sometimes give less error than compute the integral directly. In this regard, we refer to the discussion in [22].

In the setting of the TSW distribution, we have

$$\begin{array}{c} f\left(x;\lambda ,\alpha ,\beta \right)=\sum_{k=0}^{+\infty }a_{k}\upsilon_{2k+1}\left(x;\alpha ,\beta \right), \end{array}$$

where *υ*_2*k*+1_(*x*;*ζ*) denotes the pdf of the exponentiated Weibull distribution, defined with power parameter 2*k* + 1 (see [23]), i.e.,

$$\begin{array}{c} \upsilon_{2k+1}\left(x;\alpha ,\beta \right)=\left(2k+1\right)\alpha \beta x^{\beta -1}e^{-\alpha x^{\beta }}{\left(1-e^{-\alpha x^{\beta }}\right)}^{2k},\;x>0, \end{array}$$

and *υ*_2*k*+1_(*x*;*α*, *β*) = 0 if *x* ≤ 0. Further details about the exponentiated Weibull distribution can also be found in [24, 25].

### 3.5 Generalities on the moments

Let *X* be a rv having the cdf *F*(*x*;*α*, *β*, *ζ*) given by (4) (and the pdf *f*(*x*;*α*, *β*, *ζ*) given by (5)) and *ϕ*(*x*) be a function. Then, assuming that it makes mathematical sense, the expectation of *ϕ*(*X*) is obtained as

$$\begin{array}{cc} \Theta_{\phi }\left(X\right) & =E\left[\phi \left(X\right)\right]=\int_{-\infty }^{+\infty }\phi \left(x\right)f\left(x;\lambda ,\zeta \right)dx\\ & =\int_{-\infty }^{+\infty }\phi \left(x\right)\frac{\pi }{2}g\left(x;\zeta \right)\{\lambda \frac{\pi }{2}G\left(x;\zeta \right)\sin[\frac{\pi }{2}G\left(x;\zeta \right)]+\left(1-\lambda \right)\cos[\frac{\pi }{2}G\left(x;\zeta \right)]\}dx\\ & =I_{\phi }^{\left(1\right)}+I_{\phi }^{\left(2\right)}, \end{array}$$

where, by denoting *Q*_*G*_(*u*;*ζ*) the inverse function of *G*(*x*;*ζ*),

$$\begin{array}{cc} I_{\phi }^{\left(1\right)} & =\lambda \frac{\pi^{2}}{4}\int_{-\infty }^{+\infty }\phi \left(x\right)g\left(x;\zeta \right)G\left(x;\zeta \right)\sin[\frac{\pi }{2}G\left(x;\zeta \right)]dx\\ & =\lambda \frac{\pi^{2}}{4}\int_{0}^{1}\phi \left[Q_{G}\left(u;\zeta \right)\right]u\sin(\frac{\pi }{2}u)du \end{array}$$

and

$$\begin{array}{cc} I_{\phi }^{\left(2\right)} & =\left(1-\lambda \right)\frac{\pi }{2}\int_{-\infty }^{+\infty }\phi \left(x\right)g\left(x;\zeta \right)\cos[\frac{\pi }{2}G\left(x;\zeta \right)]dx\\ & =\left(1-\lambda \right)\frac{\pi }{2}\int_{0}^{1}\phi \left[Q_{G}\left(u;\zeta \right)\right]\cos(\frac{\pi }{2}u)du. \end{array}$$

These two integrals can be determined analytically, depending on the complexity of the function *ϕ*[*Q*_*G*_(*u*;*ζ*)]. In all the situations, for given baseline cdf and λ, Θ_*ϕ*_(*X*) can be calculated by the means of numerical techniques, implemented in any mathematical software.

Also, for an alternative analytical treatment, Proposition 4 implies that

$$\begin{array}{c} \Theta_{\phi }\left(X\right)=\sum_{k=0}^{+\infty }a_{k}\int_{-\infty }^{+\infty }\phi \left(x\right)\upsilon_{2k+1}\left(x;\zeta \right)dx. \end{array}$$ (8)

For practical purposes, the sum can be truncated to a large enough integer *K*, providing a suitable approximation of Θ_*ϕ*_(*X*). Some derivations of Θ_*ϕ*_(*X*) are presented in Table 1, which follow from several specific choices of *ϕ*(*x*). As an example of application, the *m*^*th*^ raw moments of a rv *X* following the TSW distribution can be derived from (8) and the *m*^*th*^ raw moments of the exponentiated Weibull distribution with power parameter 2*k* + 1 as established in [26].

**Table 1:** Specific measures and functions derived to Θ_*ϕ*_(*X*) according to the choice of *ϕ*(*x*).

Θ_*ϕ*_(*X*)	*ϕ*(*x*)
mean (μ_*_)	*x*
variance	(*x* − μ_*_)^2^
*m*^*th*^ raw moment	*x*^*m*^
*m*^*th*^ central moment	(*x* − μ_*_)^*m*^
*m*^*th*^ inverse moment	*x*^−*m*^
*m*^*th*^ logarithmic moment	[log(*x*)]^*m*^
*m*^*th*^ descending factorial moment	*x*(*x* − 1)(*x* − 2)…(*x* − *m* + 1)
*m*^*th*^ incomplete moment with respect to *t*	*x*^*m*^ if *x* ≤ *t*, and 0 elsewhere
(*m*, *q*)^*th*^ probability weighted moment	*x*^*m*^ *F*(*x*;λ, *ζ*)^*q*^
moment generating function with respect to *t*	*e*^*tx*^
characteristic function with respect to *t*	*e*^*itx*^

### 3.6 Reliability parameter

The general definition of the reliability parameter can be formulated as follows. Let *X*_1_ and *X*_2_ be two continuous rvs that can be compared based on a scenario that makes sense in a random system. Then, the corresponding reliability parameter can be defined as

$$\begin{array}{c} R=P\left(X_{2}<X_{1}\right)=\int \int_{\left\{y<x\right\}}f\left(x,y;\xi \right)dxdy, \end{array}$$

where *f*(*x*, *y*;*ξ*) denotes the joint pdf of (*X*_1_, *X*_2_), with *ξ* as parameter(s) vector. Details and applications of *R* in a concrete setting can be found in [27, 28], and the references therein.

The following result concerns the expression of *R* for the TS-G family in a specific setting.

**Proposition 5** *Let X*_1_ *and X*_2_ *be two independent rvs having the cdfs F*(*x*;λ_1_, *ζ*) *and F*(*x*;λ_2_, *ζ*), *respectively. Then, we have*

$$\begin{array}{c} R=\frac{1}{2}+\frac{1}{16}\left(\lambda_{1}-\lambda_{2}\right)\left(\pi^{2}-4\right). \end{array}$$

**Proof**. Owing to the independence of *X*_1_ and *X*_2_, and (4) and (5), and after some integral calculus, we arrive at

$$\begin{array}{cc} R & =P\left(X_{2}<X_{1}\right)=\int_{-\infty }^{+\infty }F\left(x;\lambda_{2},\zeta \right)f\left(x;\lambda_{1},\zeta \right)dx\\ & =\int_{-\infty }^{+\infty }\{\sin[\frac{\pi }{2}G\left(x;\zeta \right)]-\lambda_{2}\frac{\pi }{2}G\left(x;\zeta \right)\cos[\frac{\pi }{2}G\left(x;\zeta \right)]\}\times \\ & \frac{\pi }{2}g\left(x;\zeta \right)\{\lambda_{1}\frac{\pi }{2}G\left(x;\zeta \right)\sin[\frac{\pi }{2}G\left(x;\zeta \right)]+\left(1-\lambda_{1}\right)\cos[\frac{\pi }{2}G\left(x;\zeta \right)]\}dx\\ & =\frac{\pi }{2}\int_{0}^{1}\{\sin(\frac{\pi }{2}u)-\lambda_{2}\frac{\pi }{2}u\cos(\frac{\pi }{2}u)\}\{\lambda_{1}\frac{\pi }{2}u\sin(\frac{\pi }{2}u)+\left(1-\lambda_{1}\right)\cos(\frac{\pi }{2}u)\}du\\ & =\frac{1}{2}+\frac{1}{16}\left(\lambda_{1}-\lambda_{2}\right)\left(\pi^{2}-4\right). \end{array}$$

This ends the proof of Proposition 5.

In Proposition 5, when *X*_1_ and *X*_2_ are identically distributed, i.e., λ_1_ = λ_2_, we get *R* = 1/2. Also, Proposition 5 is useful to have a simple estimate of *R* based on estimates of λ_1_ and λ_2_. Indeed, if ${\hat{\lambda }}_{1}$ and ${\hat{\lambda }}_{2}$ are estimates of λ_1_ and λ_2_, respectively, then the plugging approach suggests the following estimate for *R*:

$$\begin{array}{c} \hat{R}=\frac{1}{2}+\frac{1}{16}\left({\hat{\lambda }}_{1}-{\hat{\lambda }}_{2}\right)\left(\pi^{2}-4\right). \end{array}$$

However, more research into the application of this formula to real-world data is needed.

## 4 Maximum likelihood estimation

Here, an inferential study of the TS-G family is proposed, estimating the parameters of the TS-G model by the maximum likelihood method.

### 4.1 The basics

The maximum likelihood method is commonly employed in parametric estimation because of its overall simplicity and the theoretical guarantees ensuring strong convergence properties on the obtained estimates. In this regard, the reader will find everything in [29]. We may also refer to [30–32] for modern applications of this method. In the context of the TS-G family, the theoretical basics of the maximum likelihood method are described below. Let *x*_1_, …, *x*_*n*_ be *n* observed values of a rv having the cdf given by (4). Then, the log-likelihood function for the parameters λ and *ζ*, supposed to be unknown, is defined as

$$\begin{array}{cc} \ell (\lambda ,\zeta ) & =\sum_{i=1}^{n}\log\left[f\left(x_{i};\lambda ,\zeta \right)\right]=n\log(\frac{\pi }{2})+\sum_{i=1}^{n}\log\left[g\left(x_{i};\zeta \right)\right]\\ & +\sum_{i=1}^{n}\log\{\lambda \frac{\pi }{2}G\left(x_{i};\zeta \right)\sin[\frac{\pi }{2}G\left(x_{i};\zeta \right)]+\left(1-\lambda \right)\cos[\frac{\pi }{2}G\left(x_{i};\zeta \right)]\}. \end{array}$$

Then, the maximum likelihood method suggests the estimates given by $\hat{\lambda }$ and $\hat{\zeta }$, which is possibly a vector of estimates, making $\ell (\hat{\lambda },\hat{\zeta })$ maximal, among all the possible values for λ and *ζ*. They are called maximum likelihood estimates (MLEs). From the ideal mathematical point of view, they are the solutions the following system of equations: ∂*ℓ*(λ, *ζ*)/∂λ = 0 and ∂*ℓ*(λ, *ζ*)/∂*ζ* = 0, with

$$\begin{array}{c} \frac{\partial \ell (\lambda ,\zeta )}{\partial \lambda }=\sum_{i=1}^{n}\frac{\frac{\pi }{2}G\left(x_{i};\zeta \right)\sin[\frac{\pi }{2}G\left(x_{i};\zeta \right)]-\cos[\frac{\pi }{2}G\left(x_{i};\zeta \right)]}{\lambda \frac{\pi }{2}G\left(x_{i};\zeta \right)\sin[\frac{\pi }{2}G\left(x_{i};\zeta \right)]+\left(1-\lambda \right)\cos[\frac{\pi }{2}G\left(x_{i};\zeta \right)]} \end{array}$$

and

$$\begin{array}{cc} \frac{\partial \ell (\lambda ,\zeta )}{\partial \zeta } & =\sum_{i=1}^{n}\frac{\partial g(x_{i};\zeta )}{\partial \zeta }\frac{1}{g(x_{i};\zeta )}+\\ & \frac{\pi }{2}\sum_{i=1}^{n}\frac{\partial g(x_{i};\zeta )}{\partial \zeta }\frac{\left(2\lambda -1\right)\sin[\frac{\pi }{2}G\left(x_{i};\zeta \right)]+\frac{\pi }{2}\lambda G\left(x_{i};\zeta \right)\cos[\frac{\pi }{2}G\left(x_{i};\zeta \right)]}{\lambda \frac{\pi }{2}G\left(x_{i};\zeta \right)\sin[\frac{\pi }{2}G\left(x_{i};\zeta \right)]+\left(1-\lambda \right)\cos[\frac{\pi }{2}G\left(x_{i};\zeta \right)]}. \end{array}$$

In most of the cases, the analytical expressions for $\hat{\lambda }$ and $\hat{\zeta }$ seem not possible. However, for given baseline cdf and λ, they can be approximated numerically by iterative techniques. Common routines are the optim function of the R software or PROC NLMIXED of the SAS (Statistical Analysis System) software. Also, one can determine the standard errors (SEs) of the MLEs which follow from the inverse of the observed information matrix. By assuming that *ζ* contains several parameters, say *m*, this observed information matrix is defined by $J={\left\{-\partial^{2}\ell \left(\hat{\lambda },\hat{\zeta }\right)/\partial \psi_{i}\partial \psi_{j}\right\}}_{i,j=1,\ldots ,m+1}$, where *ψ*_1_ = λ, and *ψ*_1+*r*_ denotes the *r*^*th*^ component of *ζ*. From the SEs, one can construct asymptotic confidence intervals of the parameters, among others.

A statistical aspect of the TS-G family that is not investigated in this study is the identifiability. Numerical experiments show no particular problem on this property, but the rigorous theory remains to be developed with precise mathematical tools.

The rest of the study is devoted to the empirical and real life applications of the TS-G model with the consideration of the MLEs of the parameters.

### 4.2 Simulation

Here, we illustrate the practical aspect of the MLEs in the setting of the TSW model, i.e., based on the TSW distribution presented in Subsection 2.3. More precisely, we propose a graphical simulation approach illustrating the numerical behavior of the MLEs $\hat{\lambda }$, $\hat{\alpha }$ and $\hat{\beta }$, of λ, *α* and *β*, respectively. The R software is used in this regard.

**Fig 4:**
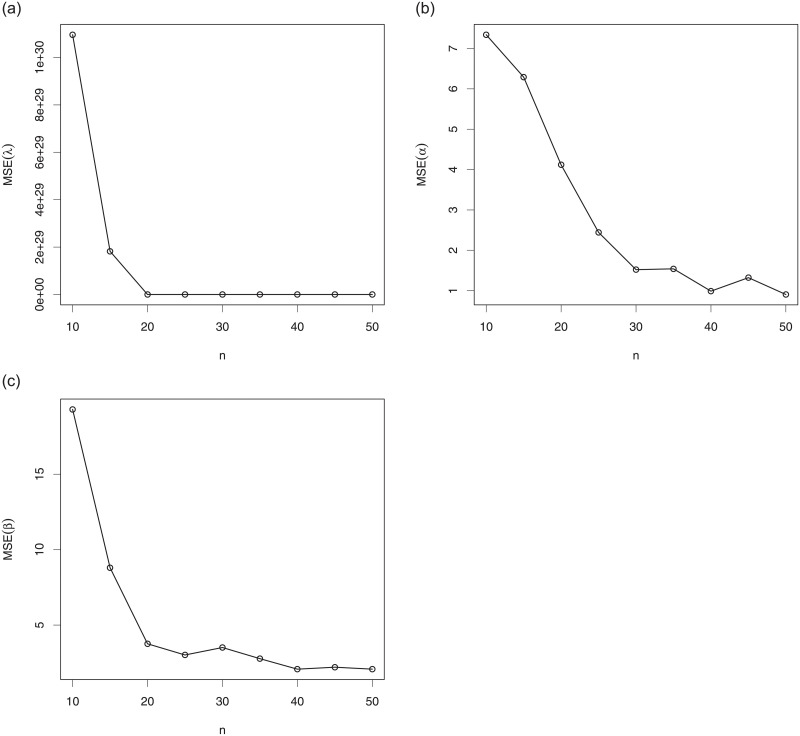
Plots of the empirical MSEs of the TSW model parameters for $S_{1}:\left(\lambda =0.3,\alpha =3,\beta =5\right)$ for (a) λ, (b) *α* and (c) *β*.

**Fig 5:**
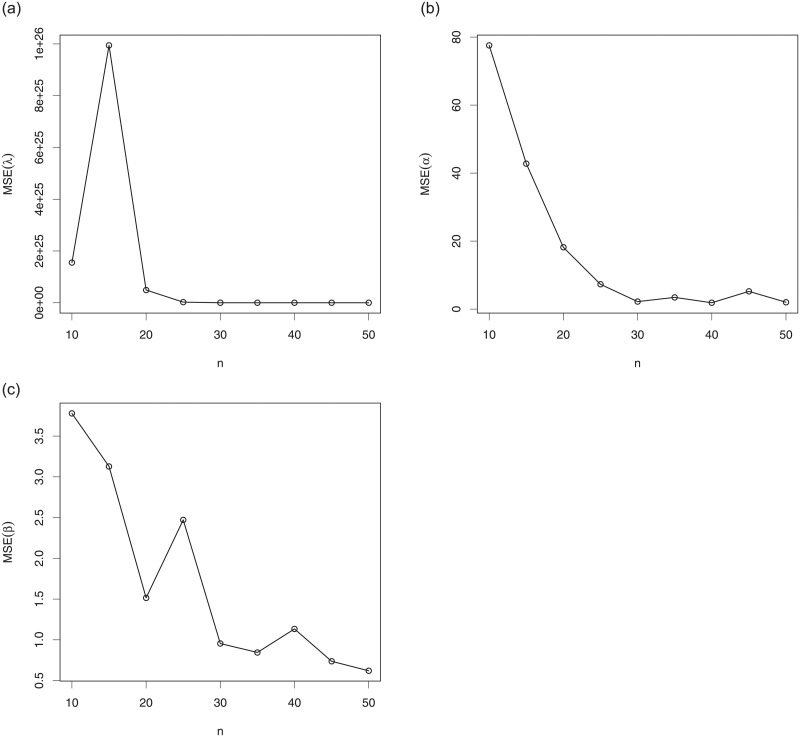
Plots of the empirical MSEs of the TSW model parameters for $S_{2}:\left(\lambda =0.1,\alpha =3.5,\beta =4.5\right)$ for (a) λ, (b) *α* and (c) *β*.

We proceed as follows. We generate *N* = 3000 samples (*x*_1_, …, *x*_*n*_) of size *n* = 10 to 50 from a rv following the TSW distribution with the two following sets of parameters: $S_{1}:\left(\lambda =0.3,\alpha =3,\beta =5\right)$ and $S_{2}:\left(\lambda =0.1,\alpha =3.5,\beta =4.5\right)$. We also calculate the empirical mean squared errors (MSEs) of the MLEs defined as, for *h* = λ, *α*, *β*,

$$\begin{array}{c} \hat{MSE_{h}}=\frac{1}{N}\sum_{i=1}^{N}{\left({\hat{h}}_{i}-h\right)}^{2}, \end{array}$$

where the index *i* refers to the *i*^*th*^ generated samples. The results of this simulation study are presented in Figs 4 and 5 for $S_{1}$ and $S_{2}$, respectively.

As a prime observation, we see that, in all the situations, when the sample size increases, the empirical MSEs approach the axis *y* = 0. This illustrates the “numerical convergence” of the MLEs to the true values of the parameters.

## 5 Applications

Thanks to its desirable flexible properties, the TSW model aims to be applied in concrete scenarios, such as the fit of real life data. We share this finding by considered the two following well-referenced real life data sets.

“The first data set”. The first data set finds its source in [28]. It contains the tensile strength (with unit in GPa) for single carbon fibers. This data set is given by: {0.312, 0.314, 0.479, 0.552, 0.700, 0.803, 0.861, 0.865, 0.944, 0.958, 0.966, 0.997, 1.006, 1.021, 1.027, 1.055, 1.063, 1.098, 1.140, 1.179, 1.224, 1.240, 1.253, 1.270, 1.272, 1.274, 1.301, 1.301, 1.359, 1.382, 1.382, 1.426, 1.434, 1.435, 1.478, 1.490, 1.511, 1.514, 1.535, 1.554, 1.566, 1.570, 1.586, 1.629, 1.633, 1.642, 1.648, 1.684, 1.697, 1.726, 1.770, 1.773, 1.800, 1.809, 1.818, 1.821, 1.848, 1.880, 1.954, 2.012, 2.067, 2.084, 2.090, 2.096, 2.128, 2.233, 2.433, 2.585, 2.585}.
“The second data set”. The second data set, often called breaking stress of carbon fibers data set, was used by [33]. This data set is given by: {3.70, 2.74, 2.73, 2.50, 3.60, 3.11, 3.27, 2.87, 1.47, 3.11, 3.56, 4.42, 2.41, 3.19, 3.22, 1.69, 3.28, 3.09, 1.87, 3.15, 4.90, 1.57, 2.67, 2.93, 3.22, 3.39, 2.81, 4.20, 3.33, 2.55, 3.31, 3.31, 2.85, 1.25, 4.38, 1.84, 0.39, 3.68, 2.48, 0.85, 1.61, 2.79, 4.70, 2.03, 1.89, 2.88, 2.82, 2.05, 3.65, 3.75, 2.43, 2.95, 2.97, 3.39, 2.96, 2.35, 2.55, 2.59, 2.03, 1.61, 2.12, 3.15, 1.08, 2.56, 1.80, 2.53}.

In addition, seven successful models are considered for comparison, also defined as extended or modified versions of the Weibull model and having two, or three or four tuning parameters. Namely, we consider the four-parameter generalized modified Weibull (GMW) model by [34], four-parameter Kumaraswamy Weibull (KW) model by [35], four-parameter beta Weibull (BW) model by [6], four-parameter odd log-logistic modified Weibull (OLLMW) model by [36], three-parameter transmuted Weibull (TW) model by [37], three-parameter modified Weibull (MW) model by [38] and the former two-parameter sine Weibull (SW) model by [3].

As criteria of goodness-of-fits to compare these models, we chose the Cramér-Von Mises (CVM), Anderson-Darling (AD) and KS statistics, with the corresponding KS p-values. Also, the AIC is calculated. For the use of the AIC in applied frameworks, one may refer to [39–41]. The global rule is the following ones. The smaller the values of the CVM, AD, KS statistics and AIC, and the larger the values of the KS p-values, the better the fit of the corresponding model to the considered data. The R software is used.

Tables 2 and 3 list the values of the CVM, AD, KS with p-value, and the MLEs and their corresponding SEs of the models parameters for the first and second data sets, respectively.

**Table 2:** CVM, AD, KS with p-value, MLEs and SEs for the first data set.

Model	CVM	AD	KS	p-value			MLEs (SEs)	
TSW	0.0152	0.1322	0.0385	1.0000	0.7896	0.4807	2.4826	-
(λ, *α*, *β*)					(0.1687)	(0.1702)	(0.4224)	-
GMW	0.0184	0.1649	0.0421	0.9960	4.5031	0.4927	0.3401	0.8561
(*a*, *α*, *γ*, λ)					(9.1201)	(0.7698)	(1.0690)	(0.2833)
KW	0.0226	0.1984	0.0475	0.9977	0.7268	0.1621	1.0308	3.5369
(*α*, *β*, *γ*, *θ*)					(0.0052)	(0.0186)	(0.0218)	(0.0086)
BW	0.0256	0.2217	0.0480	0.9973	0.3585	3.7827	0.7813	5.7953
(*α*, *β*, *γ*, *θ*)					(1.9772)	(1.2906)	(0.4103)	(18.9342)
OLLW	0.0228	0.1664	0.04675	0.9982	0.0729	0.5845	0.0146	22.3637
(*α*, *β*, *γ*, *θ*)					(0.1025)	(0.1487)	(0.0384)	(29.5884)
TW	0.0428	0.3266	0.3145	0.0000	2.7732	1.4508	-0.5636	-
(*α*, *β*, λ)					(0.4919)	(0.1420)	(0.4269)	-
MW	0.0195	0.1733	0.0431	0.9995	0.0180	0.1892	3.3740	-
(*α*, *β*, *θ*)					(0.0609)	(0.0780)	(0.5227)	-
SW	0.0236	0.2076	0.0442	0.9902	0.1291	3.0852	-	-
(*α*, *β*)					(0.0275)	(0.2951)	-	-

**Table 3:** CVM, AD, KS with p-value, MLEs and SEs for the second data set.

Model	CVM	AD	KS	p-value			MLEs (SEs)	
TSW	0.0554	0.3538	0.0694	0.9079	0.7440	0.0679	2.7694	-
(λ, *α*, *β*)					(0.1915)	(0.0504)	(0.5096)	-
GMW	0.0653	0.3939	0.0760	0.8394	5.4737	0.4343	0.1493	0.5167
(*a*, *α*, *γ*, λ)					(7.9525)	(0.6457)	(0.5395)	(0.1722)
KW	0.0703	0.4501	0.0825	0.7591	0.6536	0.1738	0.0664	3.8782
(*α*, *β*, *γ*, *θ*)					(0.0230)	(0.0416)	(0.0142)	(0.0171)
BW	0.0846	0.5041	0.0812	0.7761	0.1864	4.0715	0.7592	6.9449
(*α*, *β*, *γ*, *θ*)					(0.4201)	(1.2708)	(0.3673)	(2.7517)
OLLW	0.1032	0.54558	0.0780	0.8160	0.0729	0.5845	0.0146	22.3637
(*α*, *β*, *γ*, *θ*)					(0.0301)	(0.0828)	(0.0256)	(23.3981)
TW	0.1260	0.6700	0.3669	0.0000	2.9256	2.7531	-0.5906	-
(*α*, *β*, λ)					(0.4828)	(0.2294)	(0.3744)	-
MW	0.0640	0.40134	0.0795	0.7974	0.0165	0.0144	3.7146	-
(*α*, *β*, *θ*)					(0.0206)	(0.0079)	(0.4069)	-
SW	0.0863	0.4937	0.09003	0.6584	0.0165	3.1733	-	-
(*α*, *β*)					(0.0064)	(0.3016)	-	-

Tables 2 and 3 indicate that the smallest CVM, AD and KS and the largest KS p-value are for the TSW model; it is the best model with the considered criteria. In particular, it outperforms the former SW model corresponding to λ = 0. That is, we see that the parameter λ of the TSW model is estimated “far from zero”, i.e., the corresponding MLEs are $\hat{\lambda }=0.7896$ and $\hat{\lambda }=0.7440$, for the first and second data set, respectively. This points out the importance of the transformed sine technique to obtain suitable fits of these data, in comparison to the former SW model.

Tables 4 and 5 present the minus estimated log-likelihood, i.e., $-\hat{\ell }=-\ell \left(\hat{\lambda },\hat{\alpha },\hat{\beta }\right)$ for the TSW model, and AIC values of the model parameters for the first and second data set, respectively.

**Table 4:** The $-\hat{\ell }$ and AIC for the first data set.

Distribution	$-\hat{\ell }$	AIC
TSW	48.4389	102.8779
GMW	48.7195	106.0641
KW	48.7684	105.5368
BW	48.8954	105.7908
OLLW	49.3799	106.7600
TW	48.7059	103.4118
MW	48.9583	103.9166
SW	49.5012	103.0024

**Table 5:** The $-\hat{\ell }$ and AIC for the second data set.

Distribution	$-\hat{\ell }$	AIC
TSW	85.1358	176.2717
GMW	85.3731	178.7462
KW	85.60939	179.2188
BW	85.9184	179.8368
OLLW	85.5593	179.1187
TW	85.5453	177.0907
MW	85.52304	177.0461
SW	86.6910	177.3820

According to Tables 4 and 5, since it has the lowest AIC for the two data sets, the TSW model can be considered as the best one.

**Fig 6:**
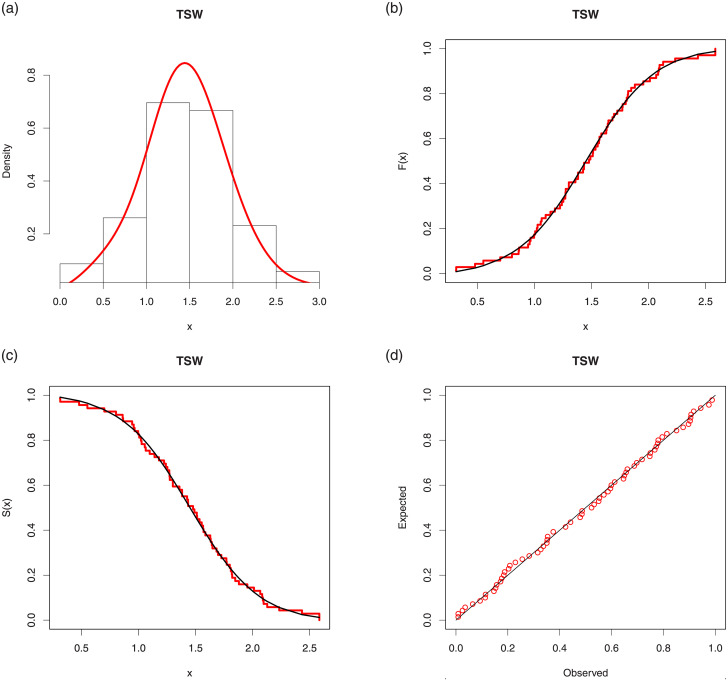
Several fits of the TSW model for the first data set: (a) estimated pdf over the histogram, (b) estimated cdf over the empirical cdf, (c) estimated sf over the empirical sf and (d) P-P plot.

**Fig 7:**
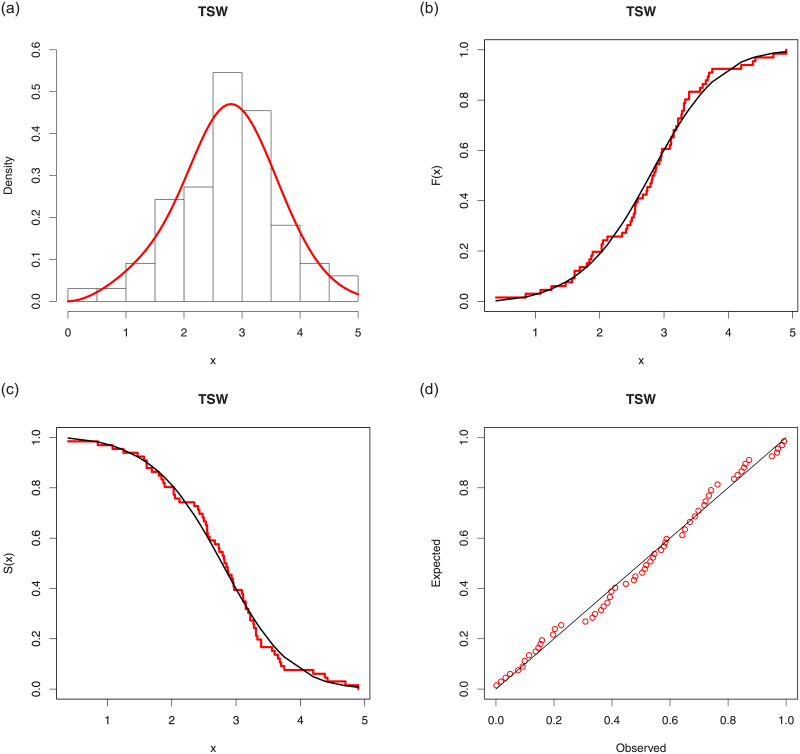
Several fits for the TSW model for the second data set: (a) estimated pdf over the histogram, (b) estimated cdf over the empirical cdf, (c) estimated sf over the empirical sf and (d) P-P plot.

We now provide a graphical visualization of the nice fitting results of the TSW model. That is, Figs 6 and 7 display several fits of the TSW model. In particular, the histograms of the both data sets are plotted, along with the curves of the corresponding estimated pdfs, i.e., $f(x;\hat{\lambda },\hat{\alpha },\hat{\beta })$, the curves of the estimated cdfs, i.e., $F(x;\hat{\lambda },\hat{\alpha },\hat{\beta })$, are plotted over the ones of the corresponding empirical cdfs of the data, the curves of the estimated sfs, i.e., $S(x;\hat{\lambda },\hat{\alpha },\hat{\beta })$, are plotted over the curves of corresponding empirical sfs of the data, and Probability-Probability (P-P) plots are provided.

In all the graphics, we see that the red curves fit well the corresponding black curves, attesting the efficiency of the TSW model in this data fitting exercise.

## 6 Concluding remarks

Based on a new one-parameter transformation function, we provide an original extension of the Sin-G family of continuous distributions, introducing the transformed Sin-G (TS-G) family. We discuss how an additional parameter λ can enhance the flexibility of the cdf of the former Sin-G family, with nice consequences for modelling purposes. An emphasis is put on the transformed Sin Weibull (TSW) distribution, showing a high potential in the analysis and modelling of lifetime data. Some general mathematical features of the TS-G family are established. Then, a statistical approach is adopted; the maximum likelihood estimates (MLEs) for the TS-G model parameters are discussed. The TSW model is highlighted, demonstrating that it is more capable of fitting data than seven rival models, some of which have more parameters. The TS-G family can find a broader use in all areas dealing with modern data as a result of its qualities. For example, it can be used to construct models in multivariate analysis, regression, classification, and other statistical fields of importance. In addition, the transformation *T*_λ_(*x*) or $T_{\lambda }^{*}\left(x\right)$ can be used to efficiently extend other existing families of distributions. These viewpoints necessitate additional developments, which we plan to incorporate in future works.
